# Presentation of Renal Cell Carcinoma Invading into the Pulmonary Artery in the Emergency Department: Case Report

**DOI:** 10.5811/cpcem.41985

**Published:** 2025-06-15

**Authors:** Sumin Yang, Corlin Jewell

**Affiliations:** University of Wisconsin School of Medicine and Public Health, Berbee Walsh Department of Emergency Medicine, Madison, Wisconsin

**Keywords:** case report, renal cell carcinoma, point-of-care ultrasound, emergency department

## Abstract

**Introduction:**

We present a case of a renal tumor infiltrating the pulmonary arteries that was diagnosed after using point-of-care ultrasound in the emergency department (ED).

**Case Report:**

A 78-year-old female presented with non-specific symptoms of heart failure. Efficient diagnosis and management were possible after using imaging in the ED that showed renal tumor extension from her left kidney to pulmonary arteries.

**Conclusion:**

This is the first case report to our knowledge on diagnosing and managing a newly discovered renal mass in the emergency setting. For non-specific symptoms of heart failure, one should consider obtaining a point-of-care ultrasound in the ED.

## INTRODUCTION

Renal cell carcinoma (RCC) is the most common type of urogenital cancer and accounts for 90% of renal malignancies.^1^ It is also more lethal compared to other cancers of that anatomical region such as bladder or prostate cancer.^2^ When a patient presents to the emergency department (ED) with RCC, symptoms are often non-specific. These symptoms can include hematuria, abdominal pain, and weight loss, which can vary depending on the anatomical region infiltrated by the mass.^3^ Right heart involvement of RCC affects 1% of patients with the diagnosis.^4^ We present a patient who presented to the ED with right-sided heart failure due to a renal mass suspicious for RCC infiltrating into heart and pulmonary arteries. Given the rarity of such a presentation, we hope this case report adds to existing literature with the goal of expanding on how an advanced case of RCC can present subtly and how it can be managed in the ED.

## CASE REPORT

We present a 78-year-old female with past medical history of hypertension, hyperlipidemia, and 40-pack-year smoking history who was sent to the ED after an outpatient transthoracic echocardiogram (TTE) detected a previously unknown mass in her right ventricle. In the ED, the patient noted some dyspnea, weakness, and fatigue for the prior two months. She also had endorsed some constipation, hemorrhoids, cough, poor sleep, cold intolerance, and worsening bilateral lower leg edema. Her edema and dyspnea were refractory after a five-day trial of furosemide, which prompted her primary care physician to obtain an outpatient TTE. The echocardiogram report noted a significant mass in the right atrium that protruded into the right ventricle and prompted a referral to the ED the same day.

The patient was alert, in no acute distress, and hemodynamically stable during her ED stay. Her physical examination was largely benign including having normal heart sounds and breathing comfortably on room air. The only remarkable physical exam finding was 2+ bilateral lower extremity pitting edema extending to her knees. Her initial lab workup was overall unremarkable with mildly elevated B-natriuretic peptide (BNP) level of 815 picograms per milliliter (pg/mL) (reference range: <450 pg/mL) and a mildly elevated creatinine of 1.04 milligrams per deciliter (mg/dL) (0.55–1.02 mg/dL). Her electrocardiogram (ECG) showed right axis deviation, but there were no previous ECGs available for comparison.

**Image 1:**
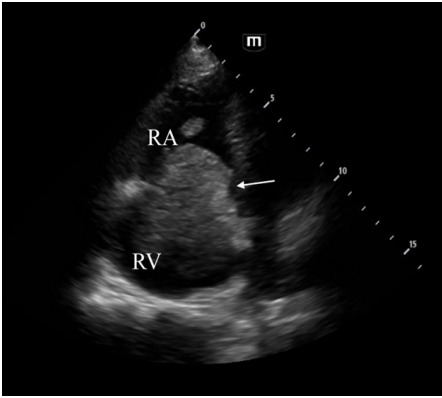
Point-of-care ultrasound image in apical 4-chamber view demonstrating dilated right ventricle (RV) with a large mass (indicated by arrow) extending from the right atrium (RA) into the right ventricle through the tricuspid valves.

**Image 2:**
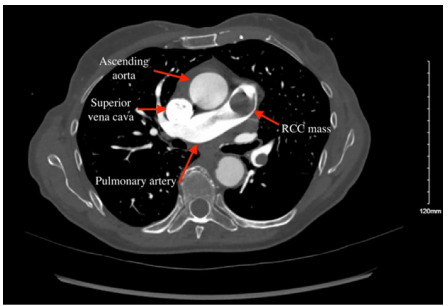
Computed tomography scan of chest in axial view of mass suspected to be renal cell carcinoma (RCC) extending into the pulmonary arteries.

A point-of-care cardiac ultrasound performed in the ED by a medical student under the resident and attending physician’s supervision showed concentric dilation of the right ventricle and a suspicious mass extending from the right atrium into the right ventricle (Image 1). Computed tomography (CT) angiogram of the chest and abdomen obtained in the ED revealed a large, heterogeneous, infiltrative mass that originated in the superior pole of the left kidney that extended into the inferior vena cava and the right atrium and ventricle, as well as into the pulmonary artery (Image 2). Maximum dimensions of the mass estimated in the right atrium were 94 millimeters (mm) in length and 37 mm in width.

Initially, the differential included acute heart failure, deep vein thrombosis, and pulmonary embolism. However, the clinical picture and imaging interpretation by a radiologist suggested a primary renal cell carcinoma with a hypervascularized tumor. The patient remained hemodynamically stable while in the ED. She was admitted to the hospitalist team for further evaluation and treatment. Interventions presented to the patient included a potential biopsy for tumor genetics to guide immunotherapy and a radical nephrectomy with thrombectomy for tumor removal. After a lengthy discussion with multiple consult teams regarding possible medical and surgical interventions, the patient decided to forgo invasive treatment and was discharged to home hospice.

*CPC-EM Capsule*

What do we already know about this clinical entity?

*Renal cell carcinoma presents with nonspecific and variable symptoms, and in rare instances, it can invade into the heart*.

What makes this presentation of disease reportable?

*A new diagnosis and initial management of renal cell carcinoma invading the pulmonary arteries in the emergency department (ED) has never been reported*.

What is the major learning point?

*Renal cell carcinoma infiltrating the pulmonary arteries was identified using point-of-care ultrasound in the ED*.

How might this improve emergency medicine practice?

*Utilizing point-of-care cardiac ultrasound can deliver better patient care in the ED in patients with nonspecific cardiopulmonary symptoms*.

## DISCUSSION

To our knowledge, this is the first case report on ED management and diagnosis of a rare presentation of renal tumor extension into the pulmonary artery. The tumor infiltration into the right ventricle explains the patient’s lack of response to diuretics due to the sheer size of the mass obstructing blood flow. Although a confirmatory biopsy was not completed for our patient, this was most likely a metastasized case of RCC given the tumor origin and presentation. Most often, RCC metastasizes to the lungs, bones, liver, adrenal glands, and lymph nodes. Prognosis of RCC has been shown to be poor for lymphatic and perinephric involvement of the metastasis rather than the size of vascular extension into the renal vein or inferior vena cava.^5^ Extension of renal tumors into the pulmonary arteries is even more rare with only two other case reports published in the literature.^6^,^7^ While this makes treatment even more challenging, it can be curable with surgical management of the primary tumor.^8^,^9^

This case highlights the critical role of point-of-care ultrasound (POCUS) in the emergency setting in diagnosis and treatment of heart failure that turned out to be a rare presentation of RCC. Relying on less time-consuming modalities for diagnosis such as laboratory markers can be enticing in the busy emergency setting. As demonstrated in our case, however, point-of-care echocardiography has been demonstrated to be more useful than BNP levels for diagnosing heart failure in the ED.^10^ In our case, it also allowed for rapid escalation of assessment with CT to further characterize the cause of the patient’s acute heart failure, which showed the hypervascularity of the tumor in detail and the physical extent of the tumor. Even though the patient decided to forgo invasive management, the rapid diagnosis made in the ED with POCUS allowed the shared decision-making process to occur without delay.

## CONCLUSION

This is the first case report that presents a rare ED presentation of a renal cell cancer mass extending into the pulmonary artery. When a patient presents with non-specific symptoms of heart failure, it is important to maintain a wide differential including new malignancy and consider obtaining a point-of-care ultrasound to guide further diagnostic and therapeutic management of patients.
